# Automatic Identification of Tool Wear Based on Convolutional Neural Network in Face Milling Process

**DOI:** 10.3390/s19183817

**Published:** 2019-09-04

**Authors:** Xuefeng Wu, Yahui Liu, Xianliang Zhou, Aolei Mou

**Affiliations:** Key Laboratory of Advanced Manufacturing and Intelligent Technology, Ministry of Education, Harbin University of Science and Technology, Harbin 150080, China

**Keywords:** tool wear monitoring, superalloy tool, convolutional neural network, image recognition

## Abstract

Monitoring of tool wear in machining process has found its importance to predict tool life, reduce equipment downtime, and tool costs. Traditional visual methods require expert experience and human resources to obtain accurate tool wear information. With the development of charge-coupled device (CCD) image sensor and the deep learning algorithms, it has become possible to use the convolutional neural network (CNN) model to automatically identify the wear types of high-temperature alloy tools in the face milling process. In this paper, the CNN model is developed based on our image dataset. The convolutional automatic encoder (CAE) is used to pre-train the network model, and the model parameters are fine-tuned by back propagation (BP) algorithm combined with stochastic gradient descent (SGD) algorithm. The established ToolWearnet network model has the function of identifying the tool wear types. The experimental results show that the average recognition precision rate of the model can reach 96.20%. At the same time, the automatic detection algorithm of tool wear value is improved by combining the identified tool wear types. In order to verify the feasibility of the method, an experimental system is built on the machine tool. By matching the frame rate of the industrial camera and the machine tool spindle speed, the wear image information of all the inserts can be obtained in the machining gap. The automatic detection method of tool wear value is compared with the result of manual detection by high precision digital optical microscope, the mean absolute percentage error is 4.76%, which effectively verifies the effectiveness and practicality of the method.

## 1. Introduction of Tool Condition Monitoring (TCM)

In milling operations, the quality of machined workpiece is highly dependent on the state of the cutting insert. Factors such as wear, corrosion, or fatigue can affect tool wear. Therefore, monitoring of tool wear in machining process has found its importance to predict tool life, reduce equipment downtime, and optimize machining parameters [1]. The detection method of tool wear can be categorized into two groups: direct and indirect method [2]. Indirect measurement is the use of sensors to measure a signal related to tool wear and obtained by analyzing the signal. Applicable signals that are widely used for tool wear measurement include acoustic emission, force, vibration, current, and power signals, etc. For example, Yao et al. [3] used the acoustic emission signal, the spindle motor current signal, and the feed motor current signal to monitor the tool wear state. Li et al. [4] used acoustic emission (AE) signals to realize the TCM and tool life prediction. Jauregui et al. [5] used cutting force and vibration signals to monitor tool wear state in the high-speed micro-milling process. Prasad et al. [6] analyzed the sound and light-emitting signals during milling and obtained the relationship between tool wear and surface roughness. However, all of these signals are heavily contaminated by the inherent noise in the industrial environment, reducing their performance [7].

Recent advances in digital image processing have suggested the machine vision should be used for TCM. In this case, the direct method of measuring tool wear has higher accuracy and reliability than the indirect method [7]. An unsupervised classification was used to segment the tool wear area through an artificial neural network (ANN) and then used to predict tool wear life [8]. Garcia-Ordas et al. [9] used computer vision technology to extract the shape descriptor of the tool wear area, combined with the machine learning model to achieve TCM. D’Addona et al. [10] used ANN and DNA-based computing, the tool wear degree was predicted based on image information extracted from the pre-processing. Alegre-Gutierrez et al. [7] proposed a method based on image texture analysis for TCM in the edge contour milling. The extracted descriptor of the tool wear area and the cutting parameters as the input parameters of wavelet neural network (WNN) model, which can predict the tool wear degree [11]. Mikolajczyk et al. [12] developed a system that can automatically measure the wear degree of the cutting edge by analyzing its image. Therefore, applying machine vision method in TCM has become more and more mature.

Since deep learning is a network structure with multiple layers of nonlinear series processing units, it is possible to omit data preprocessing and use the original data for model training and testing directly [13]. With deep learning method, enormous breakthroughs have been made in image recognition and classification [14,15,16], fault diagnosis [17,18], and medical health [19,20], etc., and CNN has become one of the research focuses in artificial intelligence algorithm directions. In the mechanical manufacturing industry, CNN can be used for monitoring the operating conditions of gearboxes, bearings, etc., to identify the faults intelligently and classify the diagnosis [21,22,23]. In the aspect of TCM, Fatemeh et al. [24] used extracted features to train and test the Bayesian network, support vector machine, K nearest neighbor regression model, and established CNN model. The experimental results show that the CNN model has a higher recognition rate precision for TCM. Zhang et al. [25] converted the original vibration signal into an energy spectrum map by wavelet packet transform, which trained and tested the energy spectrum map based on the CNN to classify the tool wear.

The above methods are used to realize real-time monitoring of the tool wear, but the research is mainly concerned with whether it is worn is qualitative monitoring, and there are relatively few studies of quantitative determination, especially for each cutting edge of the milling tool. The precision of conventional measurement methods, such as using laser measuring instruments, optical projectors, etc. are high, and so is the evaluation criterion for tool wear, but the measurement process needs to be separated from the production process with the characteristics of low efficiency, significant labor intensity, and shut-down measurements. It is challenging to meet the modern production requirements of high efficiency and automated processing [26].

The purpose of this paper is to develop a detection system that automatically recognizes tool wear types and obtains the wear value. It can obtain the wear image information of all the inserts of face milling cutter in the machining gap, and no downtime measurement, just reducing the spindle speed. In the future, the entire system can be integrated into the machine tool computer numerical control (CNC) system to achieve high automation of the machining process and measurement process. We present an on-machine measurement method to measure the tool wear. A tool wear image acquiring device installed on the working platform of the CNC machine tool is used, and the images of each cutting edge are obtained through the frame rate matching of the rotating speed. This paper draws on the VGGNet-16 network model [27] with a strong classification ability and high recognition precision, combined with the characteristics of tool wear images and the actual processing environment, to construct a CNN model to identify wear types in the machining of superalloy tools. Based on the model, the image processing method is developed according to the type of wear to improve the wear value extraction algorithm. The Roberts operator is used to locate the wear boundary and refine the edge image coarsely, thus improving the precision of automatic tool wear value detection (named ATWVD). A sample set of the tool wear image is obtained by the milling process experiments, and the tool wear value can be measured by the method. Precision and efficiency are estimated to meet the requirement of automation and precision in the milling process.

## 2. Materials and Methods

The tool wear collection device consists of the Daheng image industrial digital camera (model: MER-125-30UC, Daheng company, Beijing, China) which has a resolution of 1292 (H) × 964 (V) and a frame rate of 30 fps. Telecentric lens with a distortion rate of less than 0.08% (model: BT-2336, BTOS company, Xian, China), ring light source (model: TY63HW, Bitejia Optoelectronics company, Suzhou, China) and regulator, multi-function adjustment bracket, and a laptop computer. Three-axis vertical machining center CNC milling machine (model: VDL-1000E, Dalian machine tool company, Dalian, China), 490 R series of CVD coated carbide tools, and PVD coated carbide inserts produced by Sandvik are used in the milling of Inconel 718. The dimensions of the workpiece are 160 mm × 100 mm × 80 mm. The chemical composition of Inconel 718 material is shown in Table 1. There is no coolant during processing. The parameters of the cutting tool are shown in Table 2.

Since the CNC milling machine spindle usually does not have the function of angle control, the tool wear area cannot be accurately stopped at the captured position of the CCD, so we have to collect all the wear images of all the inserts while the spindle is rotating. Image acquisition is performed every two minutes in the milling process. It is necessary to reduce the spindle speed of the machine tool and move the face milling cutter to the coordinate measurement position. Wear images are automatically acquired by the tool wear collection device installed on the CNC machine, and then the generated image files are saved on the computer hard disk. After the automatic acquisition is completed, the inserts are removed in the process of downtime. Then the actual tool flank wear value is measured by using a precision digital optical microscope (model: Keyence VHX-1000).

The cutting parameters used for the face milling operation are shown in Table 3. There are a total of 18 possible combinations of experimental conditions (ADG, AEG, AFG, ADH, AEH, AFH, BDG, BEG, BFG, BDH, BEH, BFH, CDG, CEG, CFG, CDH, CEH, CFH) based on the given cutting parameters. Each image is labeled with a combination of cutting conditions and processing time. For example, the image “3ADG08” represents the third insert of face milling cutter, at a cutting speed of 60 m/min, a feed rate of 0.03 mm/z, a depth of cut of 0.3 mm, and a tool wear image is obtained after 8 min of machining. The above-acquired images are used as datasets.

**Figure 1:**
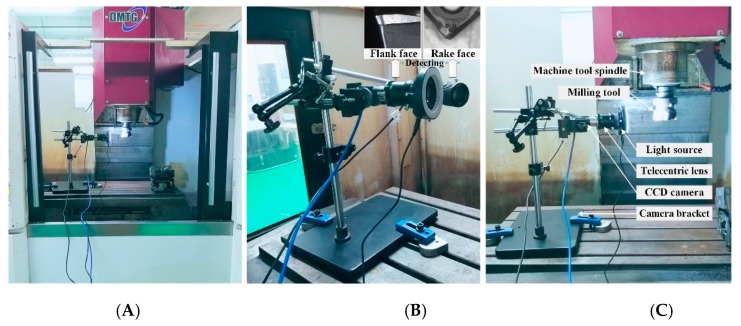
Process of matching the camera frame rate with the machine spindle speed. (**A**,**C**) Images of different numbered inserts at different speeds; (**B**) numbered picture of inserts for face milling cutter.

**Figure 2:**
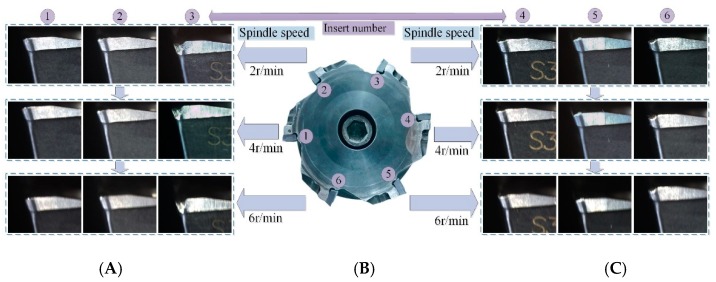
Experimental environment for milling and tool wear detection. (**A**,**C**) Actual detection images; (**B**) The overall image of camera bracket.

Each set of tests uses the same cutting parameters until the maximum flank wear of the cutting tool reaches 0.4 mm. Face milling cutters have six inserts. At the end of each set of milling tests, wear images of six inserts are acquired, and six sets of complete tool wear life cycle data are obtained. Both coated tools are used in this way. Experimental environment for milling and tool wear detection is shown in Figure 1. In the process of collecting the tool wear images, the trigger mode is a soft trigger, the exposure time is 10 ms, and the exposure mode is timed. If the spindle speed is 2 r/min, the time interval for saving the pictures is 250 ms, and the number of saved pictures is 120; if the spindle speed is 4 r/min, the time interval for saving the pictures is 150 ms, the number of saved pictures is 100, which guarantees a complete acquisition of the wear image of each cutting edge. The process of matching the camera frame rate with the machine spindle speed is shown in Figure 2. The matching between the rotational speed and the acquisition frame rate is studied, and the maximum speed of 4 r/min is obtained according to 30 fps of the industrial camera. If the spindle speed is increased, the acquired tool wear image will have different degrees of distortion. Improving the frame rate of the camera can increase the spindle speed to improve detection efficiency.

## 3. Tool Wear Types Identification Based on CNN

### 3.1. The Overall Process for Tool Wear Types Identification

**Figure 3:**
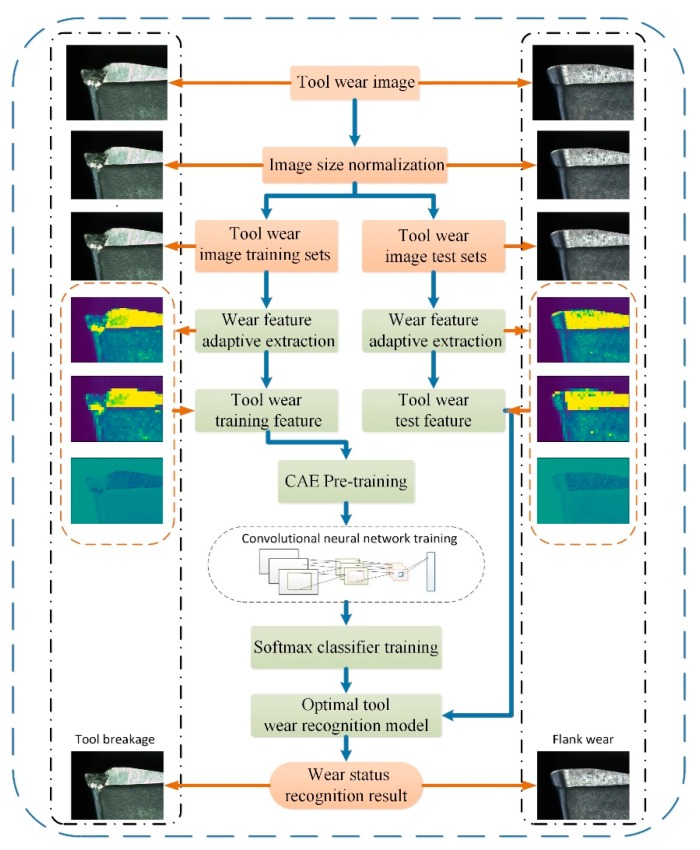
The overall process for tool wear identification.

The images of tool wear are the datasets after the image size normalization. Wear characteristics of tool wear images are extracted adaptively, some are used as the training data, and the other part is used as the test data. After the network model is pre-trained using a convolutional automated encoder (CAE), the output is set as the initial value of the CNN parameters. CNN is used to continue the training to obtain the optimal solution of the entire network parameters. Finally, the softmax classifier is used to identify and classify the wear types of milling tools. The whole network structure continuously iterates and feeds back the actual error results of the calculation. At the same time, the weight of the network is updated to make the whole network develop in the optimal direction, and the optimal wear type identification model is obtained finally. The overall process for tool wear identification is shown in Figure 3.

### 3.2. Network Structure for Tool Wear Types Identification

The CNN structure is mainly composed of a convolutional layer, a pooled layer (also referred to as a down sampling layer), and a fully connected layer. The paper constructs a recognition model of tool wear types (named ToolWearnet) based on CNN concerning a VGGNet-16 model applied to image classification and developed by the visual geometry group of Oxford University. The parameters of the network are set according to the characteristics of the tool wear identification model. The ToolWearnet model consists of 12 layers, including 11 convolutional layers and one fully connected layer, and each convolution kernel is 3 × 3 in size.

**Figure 4:**
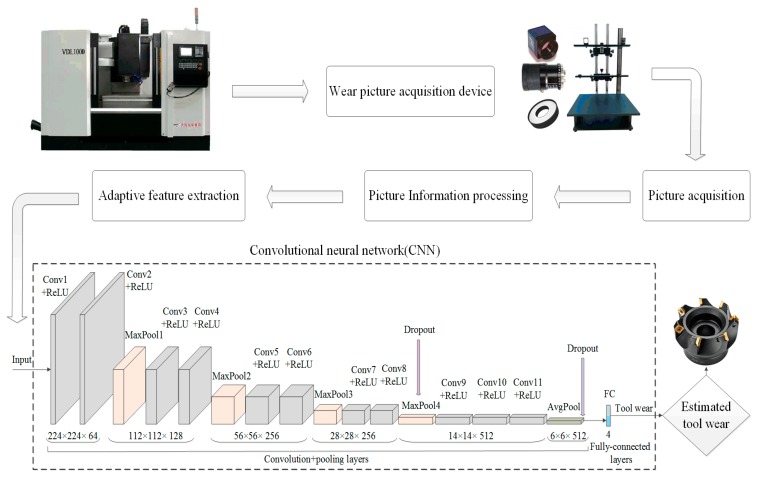
The framework of wear type identification method and network structure based on CNN.

Relative to the convolutional layer, the full connection layer has more connections, and more parameters will be generated. In order to reduce the network parameters, only one fully connected layer is set in the model, and the last pooling layer in front of the fully connected layer is set to the mean pooling layer. The kernel size of the mean pooling layer is 4 × 4. This configuration can effectively reduce network parameters while maintaining network feature extraction capabilities as much as possible. The acquired image size of the tool wear image is 256 × 256 × 3 and randomly cut into a uniform 224 × 224 resolution as the input data of the network model. The tool wear images are divided into four categories according to the wear types, and the fully connected layer is set as four types of output. The framework of tool wear identification method and network structure based on CNN are shown in Figure 4.

#### 3.2.1. Convolution Layer

The convolution operation is to use the convolution kernel to “slide” on the image at a set step size, thereby extracting features in the image to obtain feature maps. Batch normalization (BN) layer is add in the layers of Conv1, Conv3, Conv5, Conv7, Conv9, Conv10, and Conv11 to prevent over-fitting and speed up model convergence. Large-sized pictures *x_l_* (*r_l_* × *c_l_*) are operated through the convolution with the size of *x_s_* (*r_s_* × *c_s_*), the number of learned features is *k_c_*, multiple features maps *Cl_i_* (*i* = 1, 2, 3... *k_c_*) can be obtained [24] as in the following Equation (1).

(1) $$Cl_{i}=ReLu\left(W^{\left(1\right)}x_{s}+b^{\left(1\right)}\right)$$

where *W*^(1)^ and *b*^(1)^ are the weight and offset of the display unit to the hidden layer unit, respectively. *ReLu* (*x*) is the Nonlinear mapping activation function. Then the actual size of the feature map *Cl_i_* is:

(2) $$S\left(Cl_{i}\right)=\left[\left(\left(r_{l}+2\times pading-r_{s}\right)/stride\right)+1\right]\times \left[\left(\left(c_{l}+2\times pading-c_{s}\right)/stride\right)+1\right]\times k_{c}$$

where *k_c_* represents the number of convolution kernels; *padding* is the edge extension parameter; *stride* represents the step size. For example, in the CNN model of the tool wear identification, as shown in Figure 4, a tool wear image of 224 × 224 × 3 is input, a convolution kernel of 3 × 3 × 64 is used in the convolution layer Conv1 to perform convolution operation on the tool wear image. Set the step size as 1 and the edge expansion parameter as 1, and the actual size of the output feature map after convolution is 224 × 224 × 64.

#### 3.2.2. Pooling Layer

The pooling methods include maximum pooling, average pooling, and random value pooling. The dropout technology is added after the AvgPool layer and the MaxPool4 layer to reduce the complexity of interaction between neurons, so that neuron learning can obtain more robust features, improve the generalization, and avoid over-fitting of the network. Maximum pooling method and the average pooling method are used for pooling operation. Pooling kernel size is set as *p_p_* (*r_p_* × *c_p_*), and *Cl* (*r* × *c*) is the feature map obtained after the convolution operation. Multiple feature maps *p_i_* (*i* = 1,2,3…*k_p_*) obtained [25] after the pooling operation are as shown in Equations (3) and (4).

(3) $$p_{i}=\underset{r_{p}\times c_{p}}{max}\left(Cl\right)$$

(4) $$\mathrm{or}\;p_{i}=\underset{r_{p}\times c_{p}}{average}\left(Cl\right)$$

Then the actual size of the feature map pi is: Type equation here.

(5) $$S\left(p_{i}\right)=\left[\left(\left(r+2\times pading-r_{p}\right)/stride\right)+1\right]\times \left[\left(\left(c+2\times pading-c_{p}\right)/stride\right)+1\right]\times k_{p}$$

where *k_p_* represents the number of pooling kernels. For example, input a feature map of 224 × 224 × 64 in the pooling layer MaxPool1 as shown in Figure 4, pooling kernel of 2 × 2 × 128 is used for pooling operation of the tool wear images. Set the step size as 2 and the edge expansion parameter as 0, and the actual size of the output feature map after pooling operation is 112 × 112 × 128.

#### 3.2.3. Fully Connected Layer

Each neuron in the fully connected layer is fully connected to all neurons in the upper layer. After the training of the tool wear image and the corresponding label, the classification results of tool wear types are output with the recognition precision rate and loss function value of the CNN. The ToolWearnet model is constructed using the network structure described above. The size of the input tool wear image is 224 × 224. The specific parameters of each layer are shown in Table 4. The ToolWearnet model, which includes a 12-layer network structure and does not include an input layer, has 11 convolutional layers, five pooling layers, and one fully connected layer. Conv1 to Conv11 are convolutional layers, MaxPool1 to MaxPool4 are the largest pooling layer, AvgPool is the average pooling layer, and FC layer is a fully connected layer.

#### 3.2.4. Parameter Training and Fine Tuning

The training process of CNN is mainly composed of two stages: forward propagation and back propagation [28]. The network model is pre-trained by the convolutional automated encoder (CAE) and then is optimized and fine-tuned by the back propagation (BP) algorithm combined with the stochastic gradient descent (SGD) method. In the actual calculation, the batch optimization method decreases the value and gradient of the loss function in the entire training datasets. However, it is difficult to run on a PC for large data sets. The network may converge more quickly, and the above problem may be solved after applying the SGD optimization method in CNN. 

The standard gradient descent algorithm updates the parameter *θ* of the objective function *J*(*θ*) using the following formula [24]:

(6) $$\theta =\theta -\eta \nabla_{\theta }E\left[J\left(\theta \right)\right]$$

where expectation *E*[*J*(*θ*)] is the estimated value of loss function value and the gradient for all training datasets, and *η* is the learning rate.

The SGD method cancels the expected term when updating and calculating the gradient of the parameters. The formula is as follows:

(7) $$\theta =\theta -\eta \nabla_{\theta }J\left(\theta ;m^{\left(i\right)},n^{\left(i\right)}\right)$$

where (*m*^(*i*)^, *n*^(*i*)^) is the training set data pair.

When updating the model parameters, the objective function of the deep network structure has a locally optimal form, and local convergence of the network may be led by the SGD method. Increasing the momentum parameter is an improved method that can speed up the global convergence of the network. The momentum parameter updated formula is as follows [18]:

(8) $$v_{t}=\alpha v_{t-1}+\eta \nabla_{\theta }J\left(\theta ;m^{\left(i\right)},n^{\left(i\right)}\right)$$

(9) $$\theta =\theta -v_{t}$$

where *v_t_* represents the current velocity vector, and *v_t-_*_1_ represents the velocity vector of the previous moment, both having the same dimensions as the parameter *θ*; *α* is momentum factor, and *α* ϵ (0,1), where *α* is equal to 0.5.

### 3.3. Method of ATWVD Based on Tool Wear Types Identification

**Figure 5:**
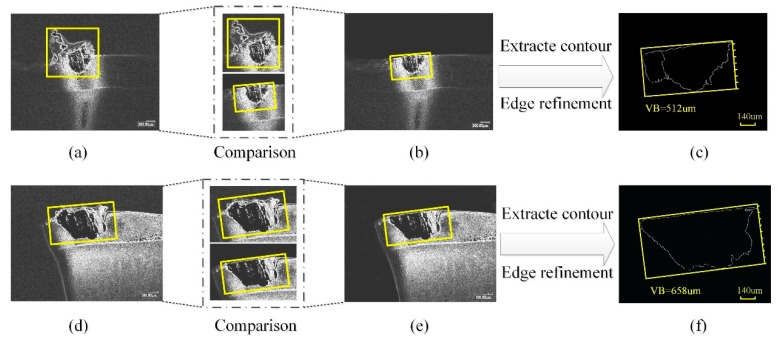
Comparison of detection methods for the value of tool wear. (**a**,**d**) Roughly processed images of adhesive wear and sheet peeling; (**b**,**e**) cropped images; (**c**,**f**) edge refined images.

The minimum bounding rectangles (MBR) method [29] is used to study the tool wear and tool breakage in milling of superalloy mentioned above. For the tool wear in the case of abrasive wear or chipping, the value of tool wear can be detected accurately, but in the case of adhesive wear and sheet peeling, the value obtained by using the MBR method directly has a significant error. This is because when the tool wear is in adhesive wear or sheet peeling, the image obtained from the camera is affected by the chips adhered on the cutting edge, as shown in Figure 5a,d, resulting in a larger rectangle surrounding the wear zone than the actual one.

In order to improve the deficiencies of the above detection methods, an ATWVD method based on tool wear types identification is proposed, the specific algorithm is as follows:

Step 1: The collected tool wear images are randomly cut into a uniform size of 224 × 224 and image size normalization was performed to achieve data normalization.
Step 2: ToolWearnet model based on CNN is used to test the tool wear images for intelligently identifying and classifying.
Step 3: After the tool wear images are normalized, the process of graying, median filter denoising, contrast stretch, segmentation boundary image using a watershed algorithm, coarse positioning using Roberts operator boundary and refining the edge image, extracting the contour of the tool wear area are conducted according to the tool wear types. If the tool wear type is adhesive wear (or the chip is sheet peeling, and the chip is bonded to the tool), the wear image is matched with the original image using image registration technology. Cropping algorithm is used to cut off the excess image to remove the contour of the chip bonded to the tool, as shown in Figure 5b,e. Finally, the area contour of effective wear is obtained, and the minimum circumscribed rectangle obtained after the edge refinement is shown in Figure 5c,f. Besides, other tool wear types are measured by directly extracting the contour of the wear area.
Step 4: According to the transformation between the image pixel value and the actual value, combined with the width of the minimum circumscribed rectangle, the actual value of tool wear value is obtained.

## 4. Results and Discussion

### 4.1. Dataset Description

**Figure 6:**
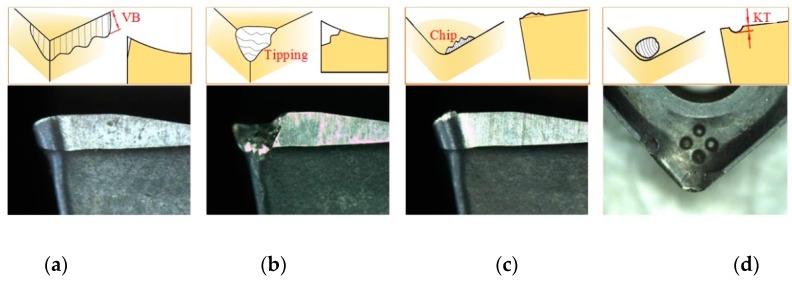
Form of tool wear and tool breakage (**a**) Flank wear; (**b**) tool breakage; (**c**) adhesive wear; (**d**) rake face wear.

The tool wear and tool failure in the milling of high superalloys are analyzed as an example. The tool wear is severe due to high hardness, excellent fatigue performance, and fracture toughness of superalloys. At a lower cutting speed, flank wear and rake face wear are the main forms of wear, as shown in Figure 6a,d; at a high-speed cutting, the heat generated causes significant chemical and adhesive wear on the tool, as shown in Figure 6c. In actual cutting, the wear form is often complicated, and multiple wears coexist. For example, flank wear and crater wear (rake face wear) can be observed simultaneously, and two wears that interact with each other will accelerate the wear of the tool and finally lead to breakage, as shown in Figure 6b. The adhesive wear at the edge will cause chips to adhere to the rake face, and finally, the sheet peeling will occur at the edge.

After the tool wear images were acquired in the milling process using the above methods, the datasets were divided into three parts: 70% as the training set; 15% as the validation set; 15% as the test set. We randomly selected two different cutting parameters and obtained twelve sets of wear images with complete tool wear life as the test set. Then, twelve sets of wear images were selected in the same way as the verification set. The remaining tool wear images were all used as the training set. The test set and training set are mutually exclusive (the test samples do not appear in the training set and are not used in the training process). The training set was used for model training, the validation set was used to select model hyper parameters, and the test set was used to evaluate model performance.

The CNN model is prone to overfitting due to its powerful fitting ability, especially when the number of datasets is small [30]. Since the number of tool wear images collected in this paper is limited, and the ToolWearnet model has more layers, more tool wear images are needed for training. Methods of rotation, flipping transformation, scaling transformation, translation transformation, color transformation, and noise disturbance are used to extend the datasets by five times. There are 8400 images in the datasets. The tool wear images mentioned above are classified according to the adhesive wear, tool breakage, rake face wear, and flank wear. After randomly cutting into a uniform, the datasets of the images were obtained. The specific number of images for each wear type is shown in Table 5.

**Figure 7:**
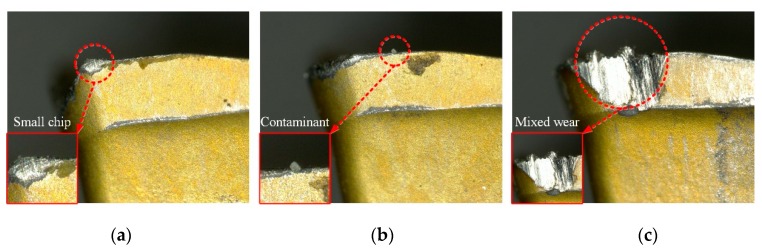
Images of the detection error cases. (**a**) adhesive wear, (**b**) flank wear, (**c**) multiple wear coexist.

The tool wear type identification is a multi-classification task. Since the tool wear types are identified in the milling machine during machining, it is likely to be affected by pollutants and fragment chips in the processing environment. Irregular chips adhere to the tool when it is adhesive wear. If the chip is small and not easily observed, it is likely that the classifier will recognize it as a rake face wear, as shown in Figure 7a. In order to reduce the probability of such errors, we collected images that are prone to recognition errors and used them as a sample set to re-train the CNN model. Similarly, if contaminants are attached to the main cutting edge of the rake face, it is likely to be recognized by the classifier as adhesive wear, as shown in Figure 7b. In order to avoid such errors, we will configure a high-pressure blowing device on one side of the machine spindle, using high pressure gas to blow away contaminants and fragment chips before performing tool wear type detection. In actual cutting, the wear form is often complicated, and multiple wears coexist. As shown in Figure 7c, it has the characteristics of adhesive wear and tool breakage, which will also cause the classifier to have recognition error. Due to the current small sample set, this mixed form of wear type has a lower occurrence probability. With the expansion of the sample set, the number of its occurrences will increase. In the future, we will further study a specialized algorithm to reduce the above error cases. 

**Figure 8:**
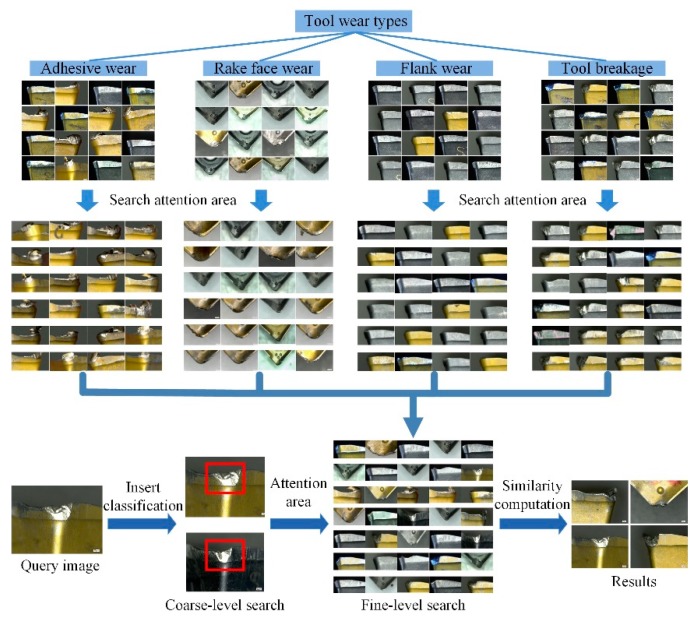
The partial images of the datasets and the process of depth search.

The CNN used in the deep learning experiment is built using the deep learning development kit provided by MATLAB2017b combined with the Caffe deep learning framework. Keyence VHX-1000 (KEYENCE company, Osaka, Japan) high precision digital optical microscope was used to test the actual tool wear value and verify the feasibility of the ATWVD method. Images manually collected by high precision digital optical microscopes are also part of the datasets. The partial images of the datasets and the process of depth search are shown in Figure 8. 

### 4.2. Evaluation Indications

After training the ToolWearnet network model, the test set was used to evaluate the precision of the ToolWearnet network model in identifying the tool wear image. We used the “precision and average recognition precision” metrics to perform the evaluation process [31], as shown in Equations (10) and (11). The ToolWearnet network model constructed in this paper has an average recognition precision (*AP*) of about 96% for tool wear types.

(10) $$precision=\;\frac{TP}{TP+FP}\;\times 100\%$$

(11) $$AP=\left[\left(\frac{1}{n}\frac{TP}{TP+FP}\right)\right]\times 100\%$$

where *TP* is the number of true positive samples in the test samples, *FP* is the number of false positive samples in the test samples, *n* is the number of categories in the test sample, *n* = 4.

In order to test the performance of the automatic tool wear value detection (named ATWVD) method based on the tool wear types in detecting the tool wear degree, the parameter error rate (*e_k_*) and mean absolute percentage error (*MAPE*) were used to evaluate, as shown in Equations (12) and (13). In this paper, the mean absolute percentage error (*MAPE*) of the ATWVD method is about 5%.

(12) $$e_{k}=\frac{\left|F_{k}-A_{k}\right|}{A_{k}}\times 100\%$$

(13) $$MAPE=\left[\left(\frac{1}{k}\overset{k}{\underset{k=1}{\sum }}e_{k}\right)\right]\times 100\%$$

where *F_k_* represents the tool wear degree detected by the ATWVD method, *A_k_* represents the wear value measured by the precision optical microscope, and *k* represents the number of measured tool wear images.

### 4.3. Analysis and Results

The initial learning rate of the training sets in the experimental phase is set to 10^−3^, the maximum number of training iterations is 10^3^, the dropout rate is 0.2, the weight attenuation is 5 × 10^−4^, and the number of batch images is 128. The algorithm of the neural network parameter optimization is stochastic gradient descent (SGD). When training this network model, the batch normalization (BN) method is used to accelerate the training of the network. Because the BN method uses the normalized method to concentrate the data at the center, it can effectively avoid the problem of the gradient disappearing. When testing this network model, the training sets are tested once every six training sessions.

#### 4.3.1. Comparison of Two CNN Models for Tool Wear Recognition

**Figure 9:**
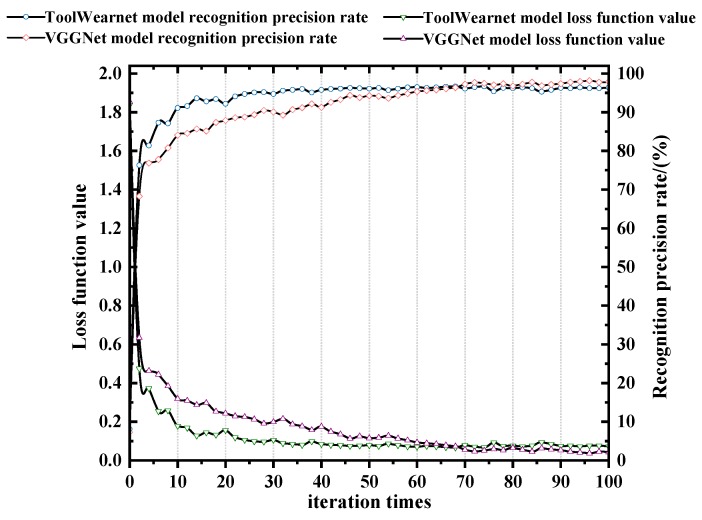
Comparison of two models testing precision rate and loss function values.

In order to reflect the reliability of the ToolWearnet network model built in this paper in tool wear types identification, and to make a reasonable evaluation of its recognition precision rate and efficiency, the VGGNet-16 network model was applied for the comparative experiment. VGGNet-16 is a CNN model developed by the VGG (visual geometry group) group of Oxford University for image classification. The VGGNet-16 network model has a 16-layer network structure with 5 maximum pooling layers, 13 convolutional layers, and 3 fully connected layers. The convolution kernels are all 3 × 3, and the pooling kernels are all 2 × 2. The first and second fully connected layers use dropout technology, and all hidden layers are equipped with ReLU layers. The model has strong image feature extraction ability. The detailed training parameters for the two models are shown in Table 6. After training and testing, different recognition precision rate and loss function values are obtained, as shown in Figure 9. The comparison of the precision rate of the recognition, training time, and recognition time of the different tool wear by the two models are shown in Table 7.

The results show that the ToolWearnet network model achieves a high precision rate at the beginning of the test quickly. In the first 24 iteration periods, the recognition precision rate of the two models are continuously increasing, and the loss function is continuously decreasing; but when the iteration period exceeds 24 times, the recognition precision rate of ToolWearnet model tends to be stable, while the recognition precision rate of the VGGNet model is continuously increasing, and its loss function is continuously decreasing. It will be stable until the iteration period exceeds 76 times. Therefore, the convergence speed of this model is faster than the VGGNet-16 network model. The VGGNet-16 model has more network layers, complex network structure, and network parameters, resulting in an astronomical calculation amount and slow convergence speed. The trend of recognition precision rate of the two models increases with the number of iterations, and the trend of the loss function decreases with the number of iterations. When the recognition precision rate and loss function of both models tend to be stable, the recognition precision rate curve of the VGGNet model is above the ToolWearnet model. Therefore, the recognition precision rate of this model is slightly lower than the VGGNet model. Compared with the ToolWearnet model, it has the deeper network depth and more feature surfaces, the feature space that the network can represent is more extensive, which will make the learning ability of the network stronger and the deeper feature information can be mined, but it will also make the network structure more complicated. The storage memory and the training time will increase.

The ToolWearnet network model has an average recognition precision rate of 96.20% for tool wear types, the highest recognition precision rate for adhesion wear, with a value of 97.27%, and the lowest recognition precision rate for flank wear, which is 95.41%. Because irregular chips adhere to the cutting edge during adhesion wear, the contour features of the wear area are most apparent, while the contour features of the flank wear are not evident due to the difference of the shape of the wear area being small. Although the recognition precision rate of the ToolWearnet network model is slightly lower than the VGGNet-16 network model, the training time and recognition time are shorter, and the hardware has low computational power requirements, which can meet the needs of slightly fast and accurate cutting in the industry.

#### 4.3.2. Tool Wear Value Detection Comparison Experiment

**Figure 10:**
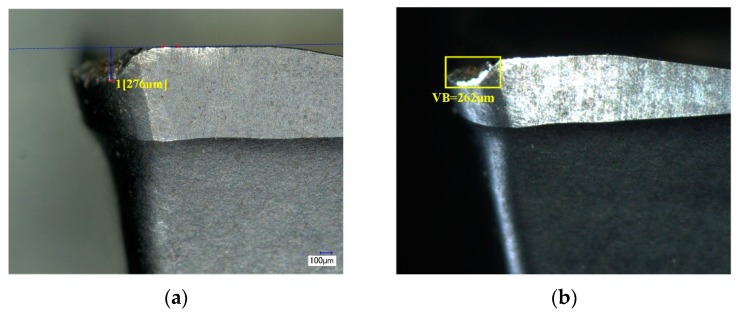
Comparison of two acquisition methods. (**a**) image is manually collected by high precision digital microscope (model: Keyence VHX-1000); (**b**) image is collected by the automatic acquisition device.

**Figure 11:**
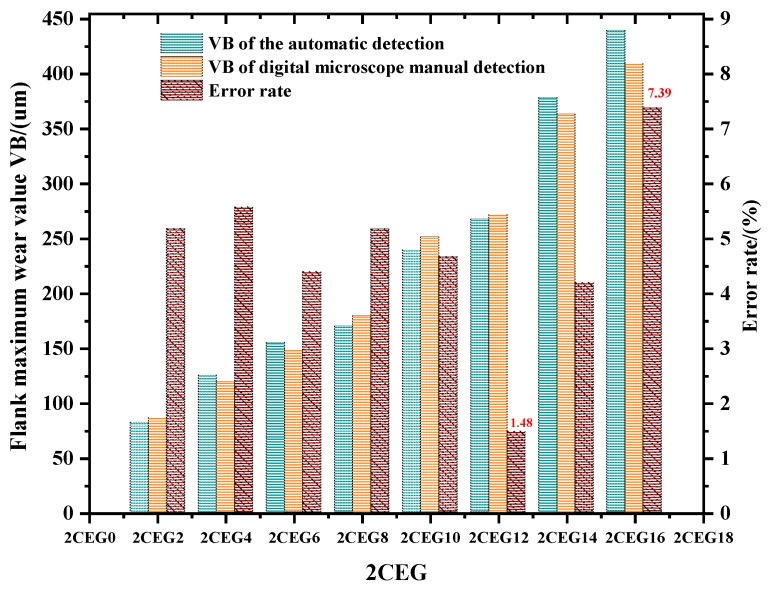
Comparison of the maximum wear value measured by the two methods and the error rate of the ATWVD method.

In the experiment, the spindle speed was matched with the frame rate of the camera, and the wear image of each insert on the face milling cutter was collected. Each acquisition took the corresponding insert directly below the label on the cutter as the starting point and sequentially numbers the six inserts in the direction while the tool rotates. The images with good quality and complete tool wear areas were acquired by filtering by the fuzzy and the insert position detection. Then, the flank wear value of each insert was obtained by the ATWVD method combined with the CNN model. After the automatic acquisition of the tool wear image, the inserts are detected by a high precision digital optical microscope to obtain the exact value of tool wear after stopping the CNC machine. The comparison of the pictures collected in two ways is shown in Figure 10. We randomly selected one set of tool wear images with complete tool wear life cycle in the test set for comparison, taking “2CEG” as an example. The maximum wear value VB of the ATWVD method detection and high precision digital microscope manual detection and the error rate of the ATWVD method were obtained, as shown in Figure 11.

The results show that the wear value detected by the ATWVD method is close to the wear value of high precision digital optical microscope manual detection, and the error rate of the wear value is in the range of 1.48~7.39% and the mean absolute percentage error (*MAPE*) is 4.76%. Therefore, the method of tool wear types automatic identification based on CNN is verified. Practicality, the process is suitable for intermittent detection of the tool, such as collecting tool information while the tool is in the tool magazine or the tool change gap. It can automatically and effectively obtain the tool wear types and wear value, which can be used to provide data for the future tool life prediction, tool selection, and tool design.

## 5. Conclusions

In this paper, an automatic recognition model of tool wear types based on CNN is proposed by using Caffe deep learning framework. The model considers the characteristics of the actual processing environment and tool wear images in milling of superalloy. The tool wear images obtained through the milling experiments of Nickel-based superalloy Inconel 718 are used as the datasets to train and test the ToolWearnet network model. The results show that the model has a robust feature extraction ability. The recognition precision rate of different wear types of high-temperature alloy tools is in the range of 95.41~97.27%, and the average recognition precision rate is 96.20%. It has the advantages of high recognition precision rate and robustness.

Furthermore, an ATWVD method is improved based on ToolWearnet network model, the value of tool wear obtained by this method is compared with the wear value detected by a high precision digital optical microscope. The error rate of this method is in the range of 1.48~7.39%, and the mean absolute percentage error is 4.76%, which proves the reliability of the method. Although the recognition precision rate of the network model is slightly lower than that of the VGGNet-16 network model, the training time and recognition time are shorter, and the network parameters are less. It can be applied to the application scenario that identifies tool wear types accurately and slightly faster with lower hardware consumption.

Practicality, the process is suitable for intermittent detection of the tool, such as collecting tool information while the tool is in the tool magazine or the tool change gap. It can automatically and effectively obtain the tool wear types and wear value, which can be used to provide data for the future tool life prediction, tool selection, and tool design. The proposed tool wear identification method can also be extended to other machining processes, such as drilling and turning. Meanwhile, future work will consider more extensive experimental data with different cutting tools, as well as extend the applications to predict tool life, optimizing the machining parameters with the method.

## Figures and Tables

**Table 1:** Chemical composition of Inconel 718 material.

Elements	C	Mn	Si	S	P	Ni	Cr	Ta
Chemical composition (%)	0.042	0.16	0.28	0.002	0.009	51.4	18.12	0.005
Mo	Al	Ti	B	Nb	Fe	Co	Cu	Pb + Bi
3.0	0.55	0.96	0.004	5.2	balance	0.2	0.11	0.001

**Table 2:** Parameters of the cutting tool.

Geometric Parameter					Material Properties		
Rake Angle, γ_0_ deg	Clearance Angle, α_0_ deg	Inclination Angle, λs deg	Corner Radius, *r* mm	Major Cutting Edge Angle, *kr* deg	Coating Materials	Basis Material	Hardness, HRC
30	6	5	0.8	75	TiAlN	C-W-Co	60

**Table 3:** Cutting parameters for face milling operation.

Cutting Speed *v_c_* (m/min)		Feed Per Tooth *f_z_* (mm/z)		Depth of Cutting *a_p_* (mm)	
**A**	**60**	**D**	0.03	G	0.3
B	109	E	0.05	H	0.5
C	148	F	0.07		

**Table 4:** Structural parameters of the ToolWearnet model.

Number	Layer Name	Size of Input Feature Maps	Size of Filters	Padding	Stride	Size of Output Feature Maps
0	Input	224 × 224 × 3	-	-	-	224 × 224 × 3
1	Conv1	224 × 224 × 3	3 × 3 × 64	1	1	224 × 224 × 64
2	Conv2	224 × 224 × 64	3 × 3 × 64	1	1	224 × 224 × 64
3	MaxPool1	224 × 224 × 64	2 × 2 × 128	2	0	112 × 112 × 128
4	Conv3	112 × 112 × 128	3 × 3 × 128	1	1	112 × 112 × 128
5	Conv4	112 × 112 × 128	3 × 3 × 128	1	1	112 × 112 × 128
6	MaxPool2	112 × 112 × 128	2 × 2 × 256	2	0	56 × 56 × 256
7	Conv5	56 × 56 × 256	3 × 3 × 256	1	1	56 × 56 × 256
8	Conv6	56 × 56 × 256	3 × 3 × 256	1	1	56 × 56 × 256
9	MaxPool3	56 × 56 × 256	2 × 2 × 256	2	0	28 × 28 × 256
10	Conv7	28 × 28 × 256	3 × 3 × 256	1	1	28 × 28 × 256
11	Conv8	28 × 28 × 256	3 × 3 × 256	1	1	28 × 28 × 256
12	MaxPool4	28 × 28 × 256	2 × 2 × 512	2	0	14 × 14 × 512
13	Conv9	14 × 14 × 512	3 × 3 × 512	1	1	14 × 14 × 512
14	Conv10	14 × 14 × 512	3 × 3 × 512	1	1	14 × 14 × 512
15	Conv11	14 × 14 × 512	3 × 3 × 512	1	1	14 × 14 × 512
16	AvgPool	14 × 14 × 512	4 × 4 × 512	2	0	6 × 6 × 512
17	FC	6 × 6 × 512	-	-	-	4

**Table 5:** Statistics of the tool wear image data.

Tool Wear Types	Training Set	Validation Set	Test Set	Total
Adhesive wear	1425	285	280	1990
Tool breakage	1455	315	305	2075
Rake face wear	1490	330	325	2145
Flank wear	1510	340	340	2190

**Table 6:** The detailed training parameters for the two models.

Network Model	Training Parameter						
	Learning Rate	Maximum Iteration Times	Dropout	Weight Attenuation	Batch Size	Optimization Algorithm	Test Interval
ToolWearnet	10^−3^	10^3^	0.2	5 × 10^−4^	128	SGD	6
VGGNet-16	10^−3^	10^3^	0.2	5 × 10^−4^	128	SGD	6

**Table 7:** The comparison of the recognition precision rate, training time, and recognition time of the two models.

Network Model	Recognition Precision Rate of Different Wear Types (%)				Training Time (h)	Recognition Time (s)
	Adhesive Wear	Tool Breakage	Rake Face Wear	Flank Wear		
VGGNet-16	97.85	97.10	96.56	95.81	32.6	174
ToolWearnet	97.27	96.35	95.78	95.41	1.8	11

## Nomenclature

*a_p_*	Depth of cutting (mm)
*f_z_*	Feed per tooth (mm/z)
*v_c_*	Cutting speed (m/min)
*padding*	Edge extension parameter
*stride*	Step size
*k_c_*	Number of convolution kernels
*k_p_*	Number of pooling kernels
*θ*	Parameter of the objective function
*η*	Learning rate
*(m^(i)^, n^(i)^)*	Training set data pair
*v_t_*	Current velocity vector
*v_t−1_*	Velocity vector of the previous moment
*α*	Momentum factor
*n*	Number of wear types
*TP*	Number of true positive samples
*FP*	Number of false positive samples
*F_k_*	Wear degree detected by the ATWVD method
*A_k_*	Wear value measured by the optical microscope
*k*	Number of measured tool wear images

## References

[1] Castro J.R., Castillo O., Melin P., Rodríguez-Díaz A. (2009). A hybrid learning algorithm for a class of interval type-2 fuzzy neural networks. Inf. Sci..

[2] Rehorn A.G., Jiang J., Orban P.E. (2005). State-of-the-art methods and results in tool condition monitoring: A review. Int. J. Adv. Manuf. Technol..

[3] Yao Y., Li X., Yuan Z. (1999). Tool wear detection with fuzzy classification and wavelet fuzzy neural network. Int. J. Mach. Tools Manuf..

[4] Li H.K., Wang Y.H., Zhao P.S., Zhang X.W., Zhou P.L. (2015). Cutting tool operational reliability prediction based on acoustic emission and logistic regression model. J. Intell. Manuf..

[5] Jauregui J.C., Resendiz J.R., Thenozhi S., Szalay T., Jacso A., Takacs M. (2018). Frequency and Time-Frequency Analysis of Cutting Force and Vibration Signals for Tool Condition Monitoring. IEEE Access.

[6] Prasad B.S., Sarcar M.M., Ben B.S. (2011). Real-time tool condition monitoring of face milling using acousto-optic emission—An experimental approach. Int. J. Comput. Appl. Technol..

[7] Garcia-Ordas M.T., Alegre-Gutierrez E., Alaiz-Rodriguez R., Gonzalez-Castro V. (2018). Tool wear monitoring using an online, automatic and low cost system based on local texture. Mech. Syst. Signal Process..

[8] Mikolajczyk T., Nowicki K., Bustillo A., Pimenov D.Y. (2018). Predicting tool life in turning operations using neural networks and image processing. Mech. Syst. Signal Process..

[9] Garcia-Ordas M.T., Alegre E., Gonzalez-Castro V., Alaiz-Rodriguez R. (2017). A computer vision approach to analyze and classify tool wear level in milling processes using shape descriptors and machine learning techniques. Int. J. Adv. Manuf. Technol..

[10] D’Addona D.M., Ullah A.M.M.S., Matarazzo D. (2017). Tool-wear prediction and pattern-recognition using artificial neural network and DNA-based computing. J. Intellig. Manuf..

[11] Ong P., Lee W.K., Lau R.J.H. (2019). Tool condition monitoring in CNC end milling using wavelet neural network based on machine vision. Int. J. Adv. Manuf. Technol..

[12] Mikolajczyk T., Nowicki K., Klodowski A., Pimenov D.Y. (2017). Neural network approach for automatic image analysis of cutting edge wear. Mech. Syst. Signal Process..

[13] Bertrand C., Jayanti M., Manuela S., Taryn J., Earley S.R., Matt J., Petrus A.E., Dubin A.P. (2010). Piezo1 and Piezo2 are essential components of distinct mechanically-activated cation channels. Science.

[14] Sa I., Ge Z., Dayoub F., Upcroft B., Perez T., McCool C. (2016). DeepFruits: A Fruit Detection System Using Deep Neural Networks. Sensors.

[15] Fuentes A., Yoon S., Kim S.C., Park D.S. (2017). A Robust Deep-Learning-Based Detector for Real-Time Tomato Plant Diseases and Pests Recognition. Sensors.

[16] Rahnemoonfar M., Sheppard C. (2017). Deep Count: Fruit Counting Based on Deep Simulated Learning. Sensors.

[17] Li S., Liu G., Tang X., Lu J., Hu J. (2017). An Ensemble Deep Convolutional Neural Network Model with Improved D-S Evidence Fusion for Bearing Fault Diagnosis. Sensors.

[18] Guo S., Yang T., Gao W., Zhang C. (2018). A Novel Fault Diagnosis Method for Rotating Machinery Based on a Convolutional Neural Network. Sensors.

[19] Dai M., Zheng D., Na R., Wang S., Zhang S. (2019). EEG Classification of Motor Imagery Using a Novel Deep Learning Framework. Sensors.

[20] Al Machot F., Elmachot A., Ali M., Al Machot E., Kyamakya K. (2019). A Deep-Learning Model for Subject-Independent Human Emotion Recognition Using Electrodermal Activity Sensors. Sensors.

[21] Chen Z., Li C., Sanchez R.V. (2015). Gearbox fault identification and classification with convolutional neural networks. Shock Vib..

[22] Zhang W., Peng G., Li C., Chen Y.H., Zhang Z.J. (2017). A New Deep Learning Model for Fault Diagnosis with Good Anti-Noise and Domain Adaptation Ability on Raw Vibration Signals. Sensors.

[23] Jing L., Zhao M., Li P., Xu X. (2017). A convolutional neural network based feature learning and fault diagnosis method for the condition monitoring of gearbox. Measurement.

[24] Fatemeh A., Antoine T., Marc T. (2018). Tool condition monitoring using spectral subtraction and convolutional neural networks in milling process. Int. J. Adv. Manuf. Technol..

[25] Zhang C., Yao X., Zhang J., Liu E. (2017). Tool wear monitoring based on deep learning. Comput. Integr. Manuf. Syst..

[26] Zhao R., Yan R., Wang J., Mao K. (2017). Learning to Monitor Machine Health with Convolutional Bi-Directional LSTM Networks. Sensors.

[27] Ren S., He K., Girshick R., Sun J. (2017). Faster R-CNN: Towards Real-Time Object Detection with Region Proposal Networks. IEEE Trans. Pattern Anal. Mach. Intell..

[28] Sainath T.N., Kingsbury B., Saon G., Soltau H., Mohamed A., Dahl G., Ramabhadran B. (2015). Deep Convolutional Neural Networks for Large-scale Speech Tasks. Neural Netw..

[29] Amit Y., Geman D. (1999). A Computational Model for Visual Selection. Neural Comput..

[30] Wen S., Chen Z., Li C. (2018). Vision-Based Surface Inspection System for Bearing Rollers Using Convolutional Neural Networks. Appl. Sci..

[31] Zhou P., Zhou G., Zhu Z., Tang C., He Z., Li W. (2018). Health Monitoring for Balancing Tail Ropes of a Hoisting System Using a Convolutional Neural Network. Appl. Sci..

